# Disparities in fine particulate matter (PM_2.5_) monitoring capacity in eastern Canada

**DOI:** 10.1088/2752-5309/ae87c0

**Published:** 2026-08-03

**Authors:** Tsz Kin Siu, Alexandra Del Favero-Campbell, Daniel G Rainham, Rachel Y-W Chang, Christopher S Greene, Kelvin C Fong

**Affiliations:** 1 Department of Earth and Environmental Sciences, Dalhousie University, 6287 Alumni Cres, Halifax, Nova Scotia B3H 4R2, Canada; 2 School of Health and Human Performance, Dalhousie University, 6230 South Street, Halifax NS B3H 1T8, Canada; 3 Department of Physics and Atmospheric Science, Dalhousie University, 1453 Lord Dalhousie Drive, Halifax B3H 2A3, Canada; 4 Department of Environmental and Occupational Health, Milken Institute School of Public Health, George Washington University, 950 New Hampshire Ave NW, Washington, DC 20037, United States of America

**Keywords:** particulate matter, air pollution monitoring, environmental justice, spatial statistics

## Abstract

Standards for air pollution are becoming more stringent. Canada will update the annual average fine particulate matter (PM_2.5_) standard to 8 *µ*g m^−3^ in 2030, which questions the adequacy of existing PM_2.5_ monitoring capacity for evaluating potential exceedances and exposure risk at community level. Although PM_2.5_ monitoring has expanded beyond regulatory monitors to include low-cost sensors, concerns remain regarding inequalities in its effective coverage and thus data representativeness for exposure estimations. Understanding the connections between PM_2.5_ monitor density, surface exposure, and neighbourhood socio-economic deprivation and health vulnerability is important for addressing compounded inequities. In this study, we applied locally adaptive kernel density estimation to calculate total PM_2.5_ monitor density from 2020 to 2024 in Ontario, Quebec, and Atlantic Canada. Local bandwidths were defined for each monitored location by considering the surrounding variability of surface PM_2.5_ concentrations and its sensitivity to distinguish exceedances. Suburban communities of Montreal, Toronto, near-border regions of Windsor and Niagara, southeast Kitchener, and remote communities in northwest Ontario had observed multiple disparities, with more pronounced socio-economic marginalization, higher surface exposure, but inadequate monitoring. Based on mixed-effects logistic regression, residential instability and situationally vulnerable populations had 25% and 54% higher odds of being deprived in PM_2.5_ monitoring capacity, respectively, after controlling for surface PM_2.5_ and emission facility density. Assessments of random-effects also suggested the importance of locally adapted strategies to account for provincial and inter-municipality variations in monitoring disparities among socio-economically marginalized groups and populations with physical or psychological disabilities. Overall, our findings inform vulnerable communities in need of monitoring expansion for precise air quality control and health risk assessments in light of the new standard.

## 1. Introduction

Fine particulate matter (PM_2.5_) poses significant health burdens even at low concentrations, driving a global re-evaluation of PM_2.5_ standards for effective pollution control and management (Shi *et al* 2016). With a diameter smaller than 2.5 *µ*m, PM_2.5_ can reach the alveoli of the lungs and enter the bloodstream, impairing pulmonary and cardiovascular function and increasing the risk of premature mortality (Manisalidis *et al* 2020). Recent studies also revealed acute and chronic impacts of PM_2.5_ exposure on mental health outcomes (Lee *et al* 2019, Roberts *et al* 2019). In Canada, PM_2.5_ is one of the criteria air contaminants (Statistics Canada 2012). The Canadian Council of Ministers of the Environment (CCME) developed the Canadian Ambient Air Quality Standards (CAAQS) in 2012 as an objective for air quality management, and is lowering the annual average limit for PM_2.5_ from 8.8 *µ*g m^−3^ to 8 *µ*g m^−3^ in 2030 (Canada, Department of the Environment and Department of Health 2025). The government has released the 2030 Emissions Reduction Plan in 2022 (Environment and Climate Change Canada (ECCC) 2022). Measurements from monitors are important for tracking progress towards new air quality goals, especially considering vulnerability across communities and populations. Therefore, identifying the spatial PM_2.5_ monitoring disparities is crucial and will inform population health risk from air pollution exposure.

Eastern Canada, comprising Ontario, Quebec, and the Atlantic provinces of New Brunswick (NB), Newfoundland and Labrador (NL), Nova Scotia (NS), and Prince Edward Island (PEI), is home to nearly half of the country’s population, most of which is concentrated within the Quebec City–Windsor Corridor (Statistics Canada 2023a). Consequently, this region experiences persistently high ambient air pollution from both traffic and industrial activities (Environment and Climate Change Canada and United States Environmental Protection Agency 2024). Moreover, PM_2.5_ can originate from direct fuel combustion and secondarily from gaseous precursors that can then disperse over long distances and affect rural areas depending on atmospheric conditions (Brook *et al* 2002, Zhang *et al* 2015). Although the Atlantic provinces generally have lower PM_2.5_ than Ontario and Quebec (Fuller-Thomson *et al* 2024), their higher proportions of vulnerable populations, including older adults and people with disabilities (Statistics Canada 2022a, 2023b), may offset the public health benefits of lower exposure. Evaluating PM_2.5_ monitoring in this region demands proper attention when considering both emissions sources and health equity. However, achieving extensive ground-level PM_2.5_ coverage is challenging due to cost and resource constraints. This has led to the deployment of ‘gold-standard’ federal equivalent method (FEM) monitors being primarily concentrated in urban areas and near major industrial sources, with only a few FEM monitors in remote regions measuring background or land-use specific concentrations (Canadian Council of Ministers of the Environment (CCME) 2019). Despite denser emissions sources in cities, a Canadian study found that rural and suburban areas could experience PM_2.5_ levels that were only about 20% lower than nearby urban centres (Wang *et al* 2021). This suggests that regulatory monitoring focus on urban sites may overlook risks elsewhere. More recently, low-cost sensors have emerged to help fill these monitoring gaps, offering accessible, real-time data for more remote communities. Findings from the U.S. suggested areas with higher poverty, unemployment, and racial minority populations had lower access to FEM monitors or low-cost PM_2.5_ sensors (deSouza and Kinney 2021, Sun *et al* 2022, Kelly *et al* 2024, Wang *et al* 2024, Haskell-Craig *et al* 2025). However, disparities in monitor distribution remain under-explored in Canada. Previous studies focused on disparities associated with PM_2.5_ concentrations, showing that lower-income, visible minority, education-marginalized groups, as well as high-risk occupations, could experience higher PM_2.5_ exposure at provincial scales (Kirby-McGregor *et al* 2023), and also tended to live closer to intra-urban traffic pollution sources (Carrier *et al* 2014). Community disparities in healthcare accessibility were also documented in Canada, especially the unmet demand of primary care providers for people with disabilities (Lavergne *et al* 2023, Wenghofer and Ransom 2023, Pucchio *et al* 2025). Adding to these, our study brings air pollution monitor capacity to the context.

Therefore, this study aims to evaluate the spatial coverage in PM_2.5_ monitoring through integrating data from FEM stations and low-cost sensors in eastern Canada under the context of 2030 CAAQS update, and to understand its relationship with neighbourhood socio-economic status (SES) and health vulnerability. We focus on two main questions:

(a) Are there PM_2.5_ monitoring gaps in areas that exceed or are at the margin of Canadian PM_2.5_ standard?
(b) Where are the socially marginalized and vulnerable communities with inadequate PM_2.5_ monitoring? Which and how are demographic characteristics associated with this inadequacy?

Findings from this study could inform placement of PM_2.5_ monitors in future measurement campaigns or continuous monitoring. Federal and provincial departments of environment or of health, in addition to community organizations supporting citizen science, could apply our methods to find monitoring gaps adapted to their objectives and plan community-level monitoring networks.

## 2. Methodology

### 2.1. Regulatory monitoring stations and low-cost sensors

To align with the current CAAQS (annual average of 8.8 *µ*g m^−3^) implemented in 2020, we assessed PM_2.5_ monitoring capacity in eastern Canada from 1 January 2020 to 31 December 2024. For FEM stations, the national air pollution surveillance programme (NAPS) manages a centralized archive of all validated hourly measurements in history (Environment and Climate Change Canada (ECCC) 2024). The latest release at the time of this study included data up to 31 December 2023. FEM data in 2024 were sourced from AirNow, an automatic reporting tool that gathers near real-time air quality information from FEM stations across the U.S. and Canada (Canadian Council of Ministers of the Environment (CCME) 2019). Additionally, PurpleAir manufactures low-cost PM_2.5_ sensors commonly used in Canada. Researchers at the University of Northern British Columbia created a platform named ‘AQMap’ storing PurpleAir sensor data in Canada for public information and research purposes (Nilson 2024). We retrieved historical hourly data of all PurpleAir sensors that were active in the six eastern Canadian provinces during the specified study period. PurpleAir sensors measure PM_2.5_ and meteorological parameters, including temperature, humidity, and air pressure, by detecting the voltage signals that vary with light scattering of ambient particles (Ardon-Dryer *et al* 2020). In this article, ‘PM_2.5_ monitors’ and ‘PM_2.5_ monitoring’ include both regulatory FEM monitoring stations and low-cost PurpleAir sensors.

### 2.2. Surface PM_2.5_ estimates

The official site selection of FEM monitors under NAPS is characterized by field-collected air samples and managerial monitoring objectives (Canadian Council of Ministers of the Environment (CCME) 2019). Satellite- and model-derived data provide a high spatial-resolution surface PM_2.5_ covering unmonitored locations, which helps quantify the spatial representativeness of each FEM monitor and PurpleAir sensor with respect to its surrounding PM_2.5_ variations. Therefore, we acquired the SatPM_2.5_ dataset, version V6.GL.02.04 (Shen 2024), published by the Atmospheric Composition Analysis Group (ACAG) at the Washington University in St. Louis. These PM_2.5_ concentration estimates were derived from a hybrid of satellite remote sensing measurements, meteorological observations, topography, emission inventory, and geophysical *a priori* information of chemical transport, using a deep convolutional neural network (Shen *et al* 2024). The model achieved a moderately high spatial cross-validation performance (global in-sample *R*^2^ = 0.91 and out-of-sample *R*^2^ = 0.86 during 2015–2019; North American in-sample *R*^2^ = 0.67 and out-of-sample *R*^2^ = 0.57 during 2001–2019). The predicted PM_2.5_ was gridded at a 0.01° × 0.01° (around 1.1 km × 1.1 km) resolution and available as annual averages spanning 1998–2023.

### 2.3. Socio-economic deprivation measures and disability population proportion

To investigate disparities by SES, we first retrieved the Canadian Index of Multiple Deprivation (CIMD), which was developed by Statistics Canada based on selected variables from 2021 Canadian Census of Population at the dissemination area (DA) level (Statistics Canada 2019). CIMD contains standardized scores of four dimensions derived from principal component analysis, namely, ‘residential instability’ (i.e. single-living, apartment renters, frequent movers, and low-income person), ‘economic dependency’ (i.e. workforce participation, unemployment, proportions of children and seniors, and government transfer payment recipients), ‘ethno-cultural composition’ (i.e. visible minorities, immigrants, foreign-born, and linguistically isolated people), and ‘situational vulnerability’ (i.e. Indigenous individuals, people with low education levels, single parents, and residents of low-value dwellings). Quintiles are obtained by ranking the DAs from the least deprived (*Q*1) to the most deprived (*Q*5) (Statistics Canada 2019). The metrics are available nationally, and regionally in Ontario, Quebec, and the Atlantic Region for eastern Canada. We used both nation-wide and province-wide scaled CIMD quintiles for mapping marginalized and under-monitored areas, and national scores were used for statistical modelling to ensure comparability across provinces. Moreover, we retrieved statistics of the 2021 census tract (CT) population with disabilities, stratified by age groups, available from the Map and Data Library Dataverse of the University of Toronto under open licence (Statistics Canada 2023c). This dataset presents physical and psychological disabilities separately based on the self-reported questions for difficulties a person perceives when performing certain daily living activities, as limited by physical functions (e.g. vision, hearing, mobility, manual dexterity, memory and learning abilities) and mental health conditions (including anxiety, depression, bipolar disorder, substance abuse, anorexia), which have lasted for at least 6 months (Statistics Canada 2023d). We calculated the percentages of total, paediatric (aged below 15), and geriatric (aged 65 or above) populations with disabilities as indicators of community-level health vulnerability.

### 2.4. Statistical analysis

#### 2.4.1. Locally adaptive kernel density estimation (KDE)

To determine whether a community is monitored by the current PM_2.5_ monitoring network, a simple approach would be either indicating whether a monitor exists or counting the number of monitors within each census unit (DA or CT). However, this method may neglect intra-unit spatial variations and the cross-boundary influence of any monitors located near the edge of a unit on its contiguous units. Areas without a monitor may share the spatial homogeneity of annual PM_2.5_ concentrations observed at the nearby monitored locations, suggesting they may already be well-represented. Instead, we characterized the coverage of PM_2.5_ monitors using KDE with locally adaptive bandwidths. In health geography, KDE was applied to estimate neighbourhood incidence of diseases, accessibility to resources and services, and distributions of pollution sources (Gatrell *et al* 1996). Specific to air pollution research, examples of KDE included analyzing spatial distributions of facility-based emissions, traffic infrastructure, and citizen-perceived hotspots (Pratt *et al* 2014, López-Aparicio *et al* 2017, Kovács and Haidu 2022, Zhang *et al* 2023), and non-spatial histogram-based distribution of pollutant concentration (Schweizer *et al* 2016, Peng *et al* 2022, Liu *et al* 2023). KDE produces a continuous surface of probability density estimates using a non-parametric kernel function (Gatrell *et al* 1996). Bandwidth controls the extent of smoothing, and the outcome of KDE is highly sensitive to the choice of bandwidths (Kuter *et al* 2011, Shi *et al* 2019). Locally adaptive bandwidths can capture spatial heterogeneity of the point pattern to optimize the final estimates (Carlos *et al* 2010, Shi 2010). For instance, researchers used night-time city lights as a proxy of local bandwidths for the KDE of alcohol exposure (Carlos *et al* 2010).

In this study, we specified bandwidths for each PM_2.5_ monitor based on the ACAG’s model-derived surface PM_2.5_ levels. We aggregated the 3 year average of annual mean predictions from 2021 to 2023. Using a 3 year average has several advantages: first, it is consistent with the statistical definition of annual PM_2.5_ limit in CAAQS; second, it avoids single-year biases; third, this period covers the two recent active wildfire years (2021 and 2023) in eastern Canada. For monitored locations below the current PM_2.5_ standard (8.8 *µ*g m^−3^), we searched for the shortest distance where a 10% absolute difference in PM_2.5_ concentration took place. Additionally, we made the bandwidth selection more sensitive near the margins (i.e. 7.2–8.8 *µ*g m^−3^) of the updated level in 2030 (8 *µ*g m^−3^), such that the searching stopped if it encountered a pixel exceeding or dropping below 8 *µ*g m^−3^ before catching the 10% gradient. For monitored locations with CAAQS exceedances, we used a more stringent criterion (5% absolute difference), assuming highly polluted areas required denser monitoring to calibrate exposure risks. Moreover, we followed Carlos *et al* (2010) to set a constraint for the maximum bandwidth, when the gradient of surrounding PM_2.5_ remained steady. Centred on each monitor’s grid, the search window expanded until it reached the upper bound of the 1.5 interquartile range rule for identifying outliers among all distances to the midpoint between a monitor and its nearest monitor. We used Euclidean distance for all distance calculations. Supplementary equation S1 provides details of the bandwidth selection process.

We implemented KDE using the ‘densityAdaptiveKernel’ function of the ‘spatstat’ package (version 3.0–5) in the R statistical computing software environment (Baddeley *et al* 2015). It considers edge-effect correction and applies the Gaussian kernel function (equation S2) (Diggle 1985, Carlos *et al* 2010, Shi 2010, Davies *et al* 2017), accelerated by a spatial partitioning technique (Davies and Baddeley 2018). In addition, accessible PM_2.5_ information not only depends on locations but also operational status of the sensors (Sun *et al* 2022). Some low-cost sensors might be deployed over a short period for testing or calibration purpose, or discontinue due to degradation in measurement performance over time (deSouza *et al* 2023). To account for the temporal data availability, we calculated an ‘accessible time fraction’, the percentage of hours with valid measurements during 2020–2024, for each PM_2.5_ monitor as the weighting factor of density estimates (equation S3). Quality control and measurement validations and corrections for data on AQMap were performed and developed by Nilson *et al* (2022), which incorporated temperature and relative humidity into models and removed observations with large measurement errors between channels A and B in each PurpleAir sensor. On 0.01° × 0.01° grids, we separately implemented KDE on FEM stations and PurpleAir sensors, then summed the two as the total density.

From the KDE results, we identified DAs with zero PM_2.5_ monitor density, indicating the spatial monitoring gaps from 2020 to 2024. Among them, we highlighted unmonitored DAs with the 3 year mean modelled surface PM_2.5_ exceeding the CAAQS. We also identified those exceeding 5 *µ*g m^−3^, the recommended annual standard by the World Health Organisation (WHO) in 2021 (World Health Organization 2021), to provide a further screening of communities with pressing monitoring needs.

#### 2.4.2. Mixed-effects logistic regression model with spatial lag

To enable comparison and modelling with census data of deprivation indices and population proportion with disabilities, we aggregated the gridded (1 km × 1 km) rasters of total PM_2.5_ monitor density and the ACAG’s modelled surface PM_2.5_ with area-weighted averaging to obtain DA and CT specific estimates. To examine the associations between inadequate PM_2.5_ monitoring and marginalization in SES and health status, we fitted mixed-effects logistic regression models. Since remote DAs and CTs are sparsely populated and occupy larger areas, the area-aggregated PM_2.5_ monitor density would be consistently lower than those near urban centres. To account for this bias, we divided total PM_2.5_ monitor density by population density, which represents the availability of PM_2.5_ monitors per person living in the DA or CT. We defined the bottom-quintile indicator of this quotient as the outcome for the mixed-effects models (‘yes’ = Q1; ‘no’ = Q2-Q5). Equation (1) describes the general form of the spatial mixed-effects logistic model, where *β_0_, β_i_, µ_0j_*, and *µ_ij_* denote the fixed-effect intercept and slopes, random-effect intercepts and slopes, respectively (i.e. *j* represents the grouping variable). $\rho Wy$ is the spatial autoregressive component, in which *W* denotes the spatial weight (adjacency) matrix.

\begin{equation*}y = \ln \left( {\frac{p}{{1 - p}}} \right) = {\beta _0} + {\mu _{0j}} + \mathop \sum \limits_{i = 1}^n \left( {{\beta _i} + {\mu _{ij}}} \right){X_i} + \rho Wy + \varepsilon .\end{equation*}

Explanatory variables (*X_i_*) included national scores of the four CIMD dimensions for the DA-level model; and proportions of population with physical and psychological disabilities for the CT-level model. We repeated the CT-level model using strata of paediatric and geriatric populations. For covariates, in addition to surface PM_2.5_, we included PM_2.5_ emission facility density, which was estimated using KDE with a cross-validated bandwidth selection by mean-squared error (MSE) (Diggle 1985) and the 3 year (2021–2023) averaged facility-specific PM_2.5_ emission inventory (Environment and Climate Change Canada (ECCC) 2025) as the weighting factor. We specified random effects grouped by provinces and census metropolitan areas (CMA) respectively for the DA-level and CT-level models. Moreover, since KDE-based PM_2.5_ monitor density likely exhibits spillover patterns, a spatially lagged term of the dependent variable was added to the model to account for the spatial structure. We used Queen contiguity as the spatial weighting method, including all neighbour units around a DA or CT with shared edges and corners, because census unit boundaries are highly irregular in size and shape, which can cause inconsistency in neighbour counts and directional bias for centroid and distance-threshold based measures. Given the binary nature of the outcome variable, the spatial lag term can be interpreted as the proportion of neighbours falling within the bottom quintile of PM_2.5_ monitoring. Parameter estimations were optimized with maximum likelihood. Residuals of the fitted models were tested with global Moran’s I statistic to ensure the absence of spatial autocorrelation. We reported odds ratio (OR) with 95% confidence interval (CI) from the model results, as well as evaluating the marginal model predictions by varying CIMD scores and disability population proportions grouped by provinces.

Sensitivity analyses were conducted to demonstrate how the model results might vary by the choices of parameters and specifications. First, we compared the bandwidth selection criterion based on different percentage changes in neighbourhood PM_2.5_, along with MSE and maximum likelihood cross-validation methods to examine the differences in spatial patterns of monitor density. Second, we investigated using absolute monitor density (i.e. number of monitors per unit area) as the outcome, while putting population density as a control variable. Third, we assessed the differences in the spatial lag term by applying inverse-distance weighting (IDW) for all neighbours within 5 km and k-nearest neighbours (k-NN) with *k* = 5, respectively.

## 3. Results

### 3.1. Monitoring gaps at risk of PM_2.5_ exceedances

**Figure 1.**
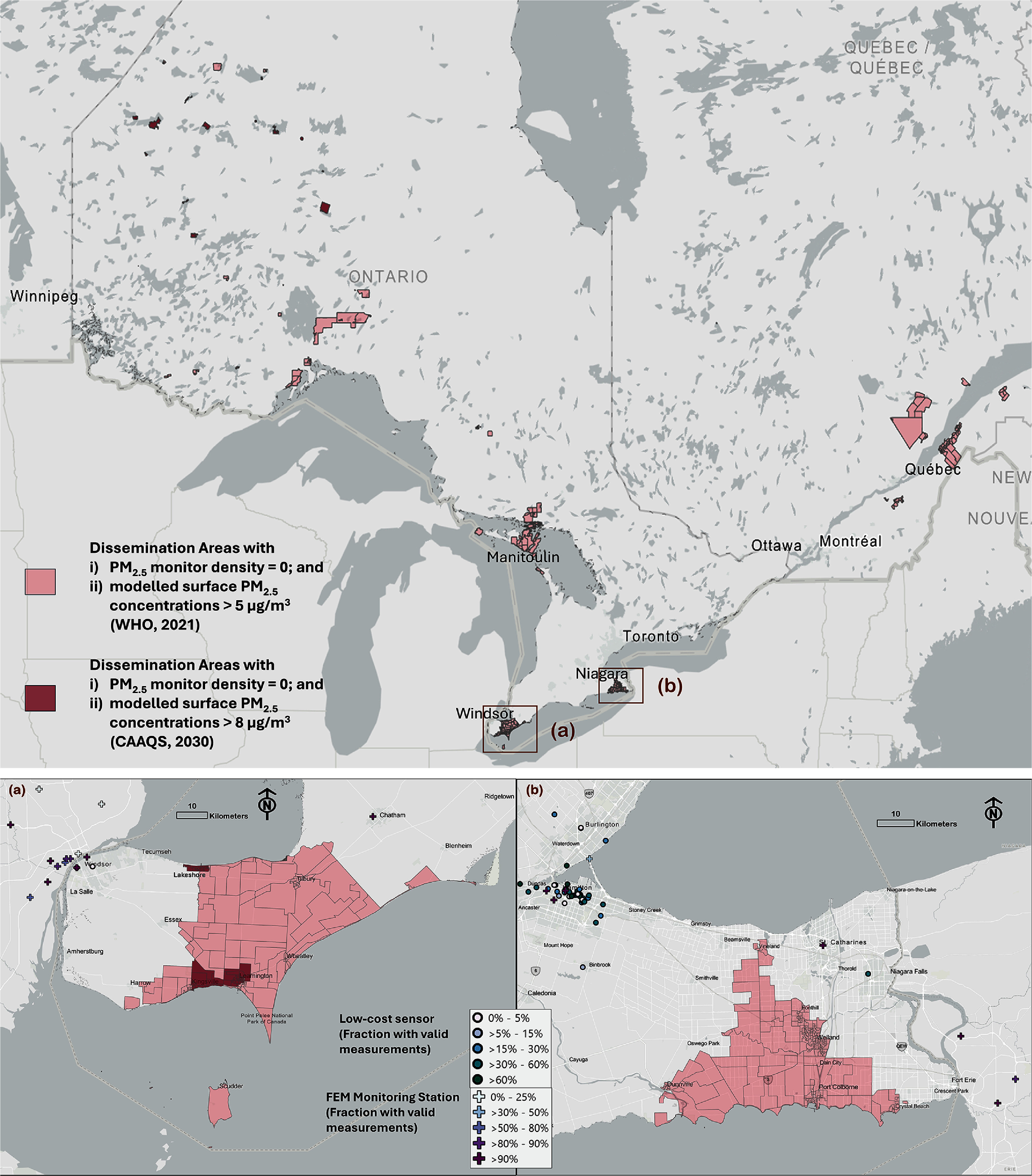
PM_2.5_ monitoring gaps (census dissemination areas with zero PM_2.5_ monitor density from the locally adaptive kernel density estimation method based on the 3 year average of 2021–2023 annual mean satellite-derived surface PM_2.5_) that exceeded the 2030 CAAQS (8 *µ*g m^−3^) and 2021 WHO’s annual guideline level for PM_2.5_ (5 *µ*g m^−3^).

Densely populated metropolitan areas, including Toronto, Montreal, Ottawa, Hamilton, Quebec City, and Halifax, had the highest PM_2.5_ monitor densities. Less populated municipalities with high industrial activities, such as Sault Ste. Marie, Sudbury, Sarnia, Saint John, and Pictou, also had concentrated monitoring (figure S1). Comparing the total monitoring density against that with only FEM monitors (figure S2), deployment of PurpleAir sensors filled data gaps in suburban and rural communities in central and northwestern Ontario, northern Quebec, and Labrador, where PM_2.5_ concentrations could reach moderate to high levels (figure S3). Clusters of monitoring gaps exceeding the CAAQS and WHO guidelines of PM_2.5_ were observed in (1) coastal suburbs near Windsor and Niagara; (2) rural communities in northwestern Ontario; (3) Manitoulin Island and its coastal neighbourhoods; (4) remote communities to the northeast, northwest, and south of Quebec City (figure 1). In particular, First Nation communities in northwestern Ontario and coastal towns of Leamington, Kingsville, and Lakeshore in Essex County exceeded the annual CAAQS in 2030. Comparing across provinces, NL had 10.07% of DAs unmonitored, followed by 3.05% for NS, 2.33% for Quebec, and 2.26% for Ontario (table S1). Monitoring gaps with highest populations, including Welland, Port Colborne, Leamington, Kingsville, and Lakeshore, are situated in Ontario. Furthermore, comparing densities of PM_2.5_ monitors and emission facilities (figure S4), we found monitoring gaps in southern Niagara concurrently having top-quintile emission sources (figure S5).

### 3.2. Disparities in PM_2.5_ monitoring among vulnerable groups

Among the four SES deprivation factors, economic dependency, residential instability, and situational vulnerability showed a significant association (*p* < 0.05) with the lowest quintile of per-capita PM_2.5_ monitor availability (table 1). Marginalizations in residential instability and situational vulnerability were associated with 25% (OR: 1.25, CI: [1.15, 1.35]) and 54% (OR: 1.54, CI: [1.31, 1.83]) higher odds of inadequate monitoring per unit increase in their principal component scores, respectively. In contrast, economic dependency had reduced odds by 43% (OR: 0.57, CI: [0.37, 0.88]). Given a generally higher total monitor density near densely populated centres (figure S1), the spatial distributions of the four CIMD dimensions could explain the differences in their regression coefficients (figure S6). For instance, economic dependency marginalization concentrated in urban areas and had a high urban-rural contrast, while situational vulnerability was more pronounced in remote areas. Incorporating random effects, Ontario distinctively had a positive association between economic dependency and inadequate monitoring, registering a 41% (CI: [31%, 52%]) increase in OR. Similarly, ethno-cultural minorities in NB faced 26% (CI: [6%, 51%]) higher odds of receiving low monitoring. Situationally vulnerable groups in Quebec had the largest increase in OR by 85% (CI: [73%, 98%]), compared to other provinces (38%–58%). DAs with higher PM_2.5_ emission density were less likely to experience inadequate monitoring (OR = 0.89, CI: [0.83, 0.95]).

**Table 1.** Association between socio-economic deprivation and low availability of PM_2.5_ monitors in the neighbourhood. The outcome variable is a binary indicator based on the quotient of KDE-derived PM_2.5_ monitor density and population density per census dissemination area (DA) level, such that ‘1’ indicates the bottom quintile (i.e. Q1, denoting low availability of PM_2.5_ monitors), ‘0’ indicates other quintiles (Q2–Q5). Mixed-effects logistic regression is used for statistical modelling, which presents the fixed effects and province-specific effects.

Covariate	Overall (Fixed) Effect		Province-specific Effect (accounting for random effects) *Odds Ratio (95% Confidence Interval)*					

	Odds Ratio (95% Confidence Interval)	*Significance ^a^*	Newfoundland & Labrador	Nova Scotia	New Brunswick	Prince Edward Island	Quebec	Ontario
*(Intercept)*	0.03 (0.01, 0.14)	***	0.04 (0.01, 0.16)	0.01 (0.00, 0.02)	0.01 (0.00, 0.04)	0.01 (0.00, 0.08)	0.03 (0.01, 0.06)	0.94 (0.76, 1.17)
Residential Instability ^b^	1.25 (1.15, 1.35)	***	1.24 (1.20, 1.29)	1.26 (1.22, 1.31)	1.29 (1.24, 1.33)	1.25 (1.20, 1.31)	1.20 (1.19, 1.22)	1.24 (1.22, 1.25)
Economic Dependency ^b^	0.57 (0.37, 0.88)	*	0.61 (0.40, 0.93)	0.40 (0.28, 0.56)	0.47 (0.31, 0.71)	0.45 (0.26, 0.77)	0.49 (0.39, 0.61)	1.41 (1.31, 1.52)
Ethno-cultural Composition ^b^	1.03 (0.87, 1.21)		1.00 (0.83, 1.21)	1.15 (0.95, 1.39)	1.26 (1.06, 1.51)	1.08 (0.85, 1.36)	0.86 (0.81, 0.91)	0.89 (0.84, 0.94)
Situational Vulnerability ^b^	1.54 (1.31, 1.83)	***	1.56 (1.26, 1.92)	1.55 (1.26, 1.90)	1.38 (1.12, 1.70)	1.58 (1.23, 2.03)	1.85 (1.73, 1.98)	1.39 (1.31, 1.47)
Modelled Surface PM_2.5_	0.88 (0.75, 1.04)		0.86 (0.74, 1.01)	1.03 (0.91, 1.16)	0.98 (0.84, 1.13)	0.98 (0.80, 1.19)	0.92 (0.85, 1.00)	0.62 (0.60, 0.63)
PM_2.5_ Emission Facility Density ^c^	0.89 (0.83, 0.95)	***	0.89 (0.87, 0.90)	0.88 (0.86, 0.90)	0.87 (0.85, 0.89)	0.88 (0.86, 0.90)	0.90 (0.90, 0.91)	0.89 (0.89, 0.90)
*(Spatial Lag of Y)*	1.09 (1.09, 1.09)	***						

a Significance: * *p* < 0.05; ** *p* < 0.01; *** *p* < 0.001

b The dimension scores were derived from principal component analysis by Statistics Canada.

c Power transformation was applied (*x^λ^*), with the exponent *λ* = 1/8, determined by optimal search with the maximum log-likelihood objective.

Fixed-effect proportions of population with physical and psychological disabilities had no significant association with PM_2.5_ monitoring patterns (table 2). Province-specific effects suggested that CTs with higher paediatric physical disability rates were likely under-monitored (OR: 1.07–1.09, CI: [1.03–1.05, 1.06–1.10]). Except for Ontario, other provinces had around 4% (CI: [1%, 8%]) higher odds of inadequate PM_2.5_ monitoring for children with psychological disabilities. Considering CMA-specific effects (tables S2–S4), all CMAs had 95% CIs of OR greater than 1 for children with physical disabilities, which also applied to Oshawa and Montreal for children with psychological disabilities. Similar observations were found in Oshawa, St. Catharines–Niagara, and Toronto for communities with both higher geriatric physical and psychological disability rates, additionally including Quebec City, Kingston, and London when limited to the older adults with psychological disabilities.

**Table 2.** Association between health status (represented by proportion of population with physical and psychological disabilities, considering strata of child and elderly populations) and low availability of PM_2.5_ monitors in the neighbourhood. The outcome variable is a binary indicator based on the quotient of KDE-derived PM_2.5_ monitor density and population density per census dissemination area (DA) level, such that ‘1’ indicates the bottom quintile (i.e. *Q*1, denoting low availability of PM_2.5_ monitors), ‘0’ indicates other quintiles (*Q*2–*Q*5). Mixed-effects logistic regression is used for statistical modelling, which presents the fixed effects and province-specific effects.

Strata	Covariate	Overall (Fixed) Effect		Province-specific Effect (accounting for random effects) *Odds Ratio (95% Confidence Interval)*				
		Odds Ratio (95% Confidence Interval)	*Significance ^a^*	Newfoundland & Labrador	Nova Scotia	New Brunswick	Quebec	Ontario

Total Population	*(Intercept)*	0.28 (0.04, 1.96)		0.25 (0.04, 1.38)	0.27 (0.05, 1.55)	0.25 (0.04, 1.46)	0.39 (0.10, 1.58)	0.26 (0.07, 0.94)
	% Pop. with Physical Disabilities	0.93 (0.52, 1.66)		0.91 (0.77, 1.08)	0.92 (0.81, 1.05)	0.79 (0.61, 1.04)	1.03 (0.94, 1.14)	0.99 (0.96, 1.03)
	% Pop. with Psychological Disabilities	1.05 (0.90, 1.21)		1.07 (0.89, 1.29)	1.11 (0.93, 1.33)	1.05 (0.86, 1.29)	1.01 (0.99, 1.03)	0.99 (0.97, 1.01)
	Modelled Surface PM_2.5_	0.75 (0.36, 1.57)		0.69 (0.27, 1.73)	0.55 (0.24, 1.26)	0.90 (0.34, 2.38)	0.80 (0.66, 0.96)	0.89 (0.75, 1.05)
	PM_2.5_ Emission Facility Density ^b^	0.63 (0.30, 1.33)		0.63 (0.24, 1.61)	0.75 (0.31, 1.82)	0.52 (0.17, 1.58)	0.56 (0.37, 0.83)	0.73 (0.54, 0.99)
	*(Spatial Lag of Y)*	1.07 (1.07, 1.08)	***					

Paediatric Population(Age < 15)	*(Intercept)*	0.05 (0.01, 0.18)	***	0.05 (0.02, 0.11)	0.05 (0.02, 0.11)	0.04 (0.02, 0.10)	0.05 (0.02, 0.09)	0.08 (0.05, 0.14)
	% Pop. with Physical Disabilities	1.06 (0.98, 1.16)		1.07 (1.04, 1.09)	1.07 (1.04, 1.09)	1.07 (1.04, 1.10)	1.07 (1.05, 1.09)	1.05 (1.03, 1.06)
	% Pop. with Psychological Disabilities	1.04 (0.98, 1.10)		1.04 (1.01, 1.08)	1.04 (1.01, 1.07)	1.04 (1.01, 1.08)	1.04 (1.02, 1.07)	1.02 (1.00, 1.04)
	Modelled Surface PM_2.5_	1.00 (0.87, 1.15)		1.01 (0.93, 1.09)	1.01 (0.93, 1.09)	1.02 (0.94, 1.10)	1.01 (0.95, 1.07)	0.95 (0.91, 1.00)
	PM_2.5_ Emission Facility Density ^b^	0.65 (0.52, 0.81)		0.64 (0.56, 0.73)	0.64 (0.56, 0.73)	0.63 (0.55, 0.72)	0.64 (0.58, 0.70)	0.70 (0.65, 0.76)
	*(Spatial Lag of Y)*	1.07 (1.07, 1.08)	***					

Geriatric Population(Age ⩾ 65)	*(Intercept)*	0.35 (0.05, 2.62)		0.34 (0.07, 1.64)	0.36 (0.07, 1.72)	0.39 (0.08, 1.90)	0.48 (0.13, 1.80)	0.25 (0.07, 0.88)
	% Pop. with Physical Disabilities	1.01 (0.55, 1.85)		1.05 (0.85, 1.31)	1.04 (0.88, 1.22)	0.94 (0.70, 1.26)	1.02 (0.96, 1.08)	1.01 (0.98, 1.04)
	% Pop. with Psychological Disabilities	1.05 (0.65, 1.69)		1.02 (0.63, 1.66)	1.09 (0.80, 1.49)	1.11 (0.66, 1.86)	0.99 (0.90, 1.09)	1.02 (0.97, 1.06)
	Modelled Surface PM_2.5_	0.60 (0.25, 1.44)		0.46 (0.16, 1.33)	0.40 (0.13, 1.20)	0.66 (0.19, 2.22)	0.78 (0.63, 0.97)	0.82 (0.69, 0.97)
	PM_2.5_ Emission Facility Density ^b^	0.57 (0.23, 1.44)		0.52 (0.16, 1.73)	0.76 (0.24, 2.42)	0.44 (0.11, 1.69)	0.53 (0.30, 0.94)	0.66 (0.43, 1.01)
	*(Spatial Lag of Y)*	1.07 (1.07, 1.08)	***					

a Significance: * *p* < 0.05; ** *p* < 0.01; *** *p* < 0.001

b Power transformation was applied (*x^λ^*), with the exponent *λ* = 1/8, determined by optimal search with the maximum log-likelihood objective.

**Figure 2.**
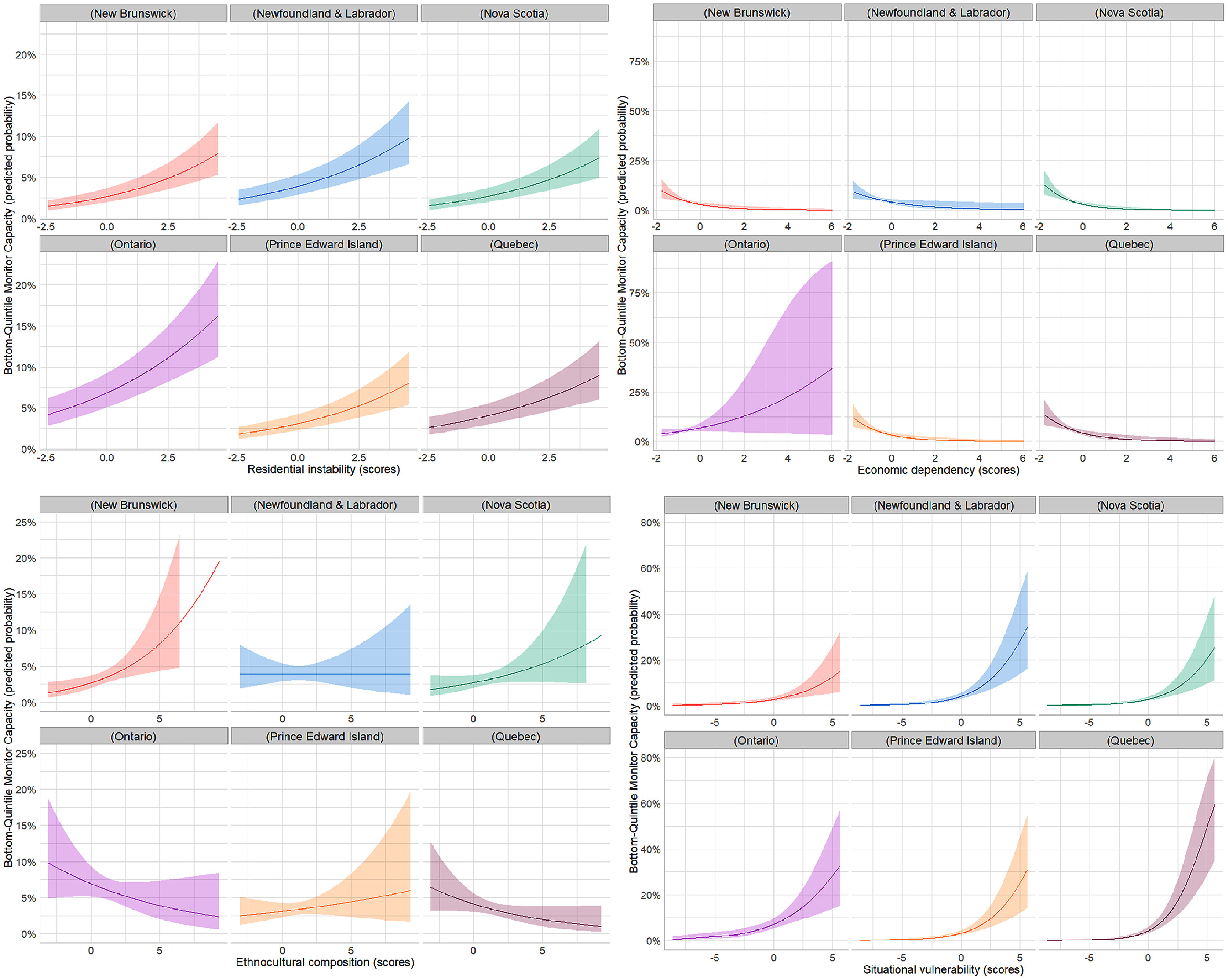
Adjusted predicted probabilities for the response (bottom-quintile per-capita PM_2.5_ monitor availability) against the four CIMD dimension scores grouped by six provinces in the mixed-effects logistic regression model, with other non-focal terms controlled at marginal means.

**Figure 3.**
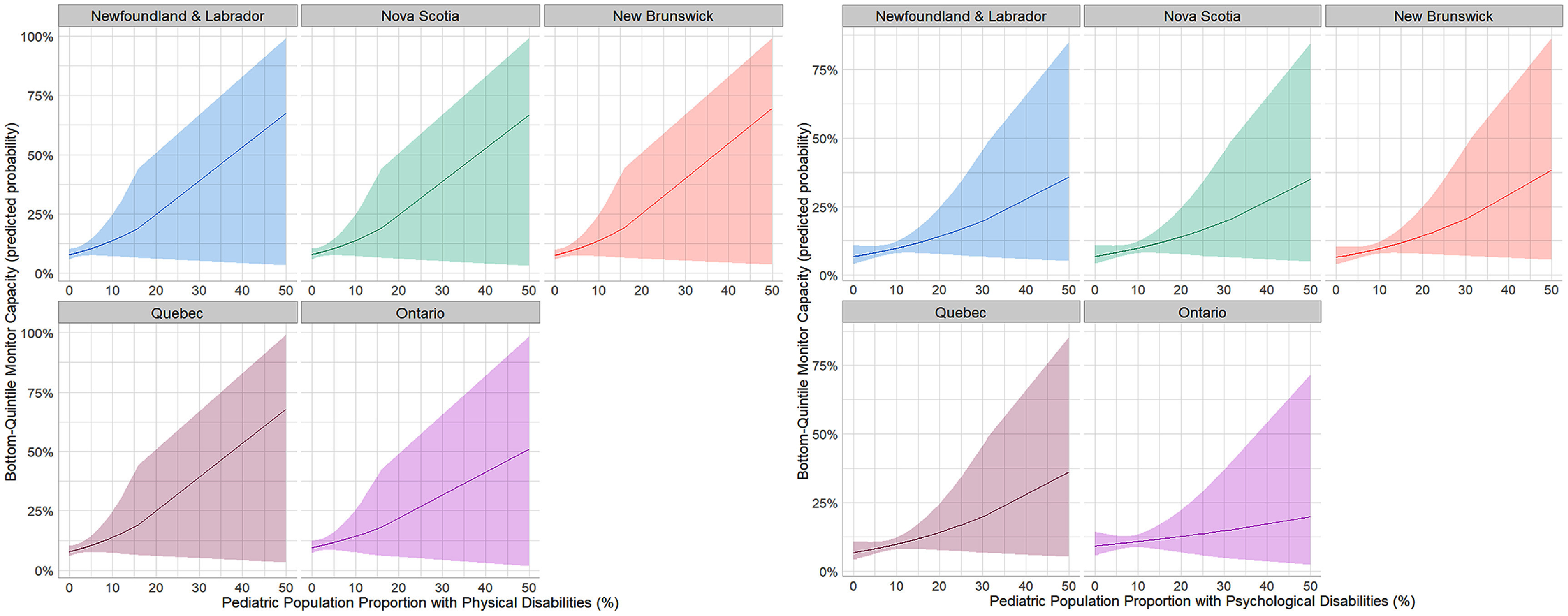
Adjusted predicted probabilities for the response (bottom-quintile per-capita PM_2.5_ monitor availability) against the proportion of paediatric population (aged under 15) with physical and psychological disabilities grouped by five provinces (P.E.I. is excluded due to no census tract) in the mixed-effects logistic regression model, with other non-focal terms controlled at marginal means.

Extending from the significant random effects in the CIMD and paediatric disability models, the predicted probabilities across the ranges of individual explanatory variables further illustrated how likely different socio-economic and health marginalization levels would have low monitor capacity (figures 2 and 3). All provinces observed consistent increases in probabilities for residential instability and situational vulnerability, with the latter having steeper slopes at high extremes of marginalization scores. In Ontario, highly residence-marginalized and economically dependent communities had over 15% and 25% chances in the bottom monitoring quintile, respectively. In Quebec, situational vulnerability observed the most significant rise in probability, exceeding 40% at high extremes. Apart from the significantly increasing trend for NB, despite the absence of statistically significance in OR, ethno-cultural marginalization in NS and PEI still observed gradually increasing curves with probabilities. CTs with a paediatric physical disability rate of approximately 20% would have probabilities reaching 25%. This also applied to those with a paediatric psychological disability rate exceeding 35%, except for Ontario.

Combined inequities in PM_2.5_ monitoring, SES deprivation, and satellite-derived PM_2.5_ exceedances were most common in suburban areas in southern Ontario and Quebec. Municipalities ranked by prioritizing the intra-urban proportions of under-monitored and high-pollution DAs, total populations living in those DAs, and degrees of CIMD marginalization underscored disadvantages in suburbs near Montreal (e.g. Blainville, Repentigny) and the northern Greater Toronto Area (e.g. Brampton) (table 3). Detailed mapping of these DAs also found marginalized communities near southeastern Windsor and Kitchener had exceedances according to the 2030 annual CAAQS (figure S7). For under-monitored CTs, southeast Kitchener with Cambridge, south-central Oshawa, southwest London with St. Thomas, and southern Niagara (Welland, Port Colborne, and Canada-U.S. border communities in Niagara Falls and Fort Erie) had relatively higher proportions of population with physical and psychological disabilities and PM_2.5_ exceedances (table S5 and figure S8). We further separated the results by paediatric and geriatric populations in supplementary information (figures S9 and S10), to reveal the health-vulnerable groups facing inadequate monitoring in specific communities.

**Table 3.** Under-monitored census communities (subdivisions) evaluated on the number of dissemination areas (DA) situated in the bottom quintile of the per-capita PM_2.5_ monitor availability (i.e. outcome of the mixed-effects logistic regression model) and with the 3 year averaged surface PM_2.5_ concentrations either (a) exceeding 8.8 *µ*g m^−3^ (i.e. high-risk area), or (b) at the margin of exceedance level in 2030 CAAQS (i.e. 7.2–8.8 *µ*g m^−3^). For each category, the rankings were obtained by prioritizing three different criteria, respectively: (1) highest proportions of under-monitored DA within the community; (2) most population living in under-monitored DA; (3) most marginalized by CIMD measures. Only subdivisions consisting of more than 10 DAs were listed, there are under-monitored remote communities, like the First Nations reserves, and full mapping details can be found in Figure S7 in the supplemental.

(a) High-risk area (exceeding 8.8 *µ*g m^−3^)									
Census Sub-Division (CSD)	Province	% DA under-monitored	Total CSD Population (‘000)	Population in under-monitored DA (‘000)	Overall Rank of CIMD (N = 42)	Rank of Residential Instability (N = 42)	Rank of Economic Dependency (N = 42)	Rank of Ethno-cultural Composition (N = 42)	Rank of Situational Vulnerability (N = 42)

(1) ^*^									
Charlemagne	Quebec	80.0	6.3	5.3	12	7	33	10	20
Lorraine	Quebec	73.3	9.5	6.4	41	42	36	35	38
Sainte-Thérèse	Quebec	51.1	24.9	12.7	22.5	4	30	18	21
**Blainville**	**Quebec**	**32.8**	**59.0**	**21.2**	**36.5**	**29**	**31**	**39**	**37**
Bois-des-Filion	Quebec	31.3	10.0	3.7	27	15	25	34	27
(2) ^*^									
Toronto	Ontario	1.2	2,761.2	70.3	10	6	2	23	31
Mississauga	Ontario	8.6	712.7	63.0	9	12	3	16	19
**Brampton**	**Ontario**	**4.4**	**650.1**	**27.6**	**6**	**10**	**4**	**22**	**15**
**Windsor**	**Ontario**	**5.0**	**226.4**	**21.2**	**7.5**	**19**	**8**	**3**	**24**
**Blainville**	**Quebec**	**32.8**	**59.0**	**21.2**	**36.5**	**29**	**31**	**39**	**37**
(3) ^*^									
Richmond Hill	Ontario	0.5	200.9	1.7	1	3	1	6	40
Laval	Quebec	0.2	429.6	0.7	2	5	10	1	16
Montréal	Quebec	0.2	1,723.3	2.4	5	9	6	26	14
**Brampton**	**Ontario**	**4.4**	**650.1**	**27.6**	**6**	**10**	**4**	**22**	**15**
**Windsor**	**Ontario**	**5.0**	**226.4**	**21.2**	**7.5**	**19**	**8**	**3**	**24**
**(b) 2030 CAAQS Exceedance-margin area (7.2–8.8 *µ*g m^−3^)**									

Census Sub-Division (CSD)	Province	% DA under-monitored	Total CSD Population (‘000)	Population in under-monitored DA (‘000)	Overall Rank of CIMD (*N* = 221)	Rank of Residential Instability (*N* = 221)	Rank of Economic Dependency (*N* = 221)	Rank of Ethno-cultural Composition (*N* = 221)	Rank of Situational Vulnerability (*N* = 221)

(1) ^*^									
Hawkesbury	Ontario	100.0	9.8	9.8	9	18	79	14	39
Leamington	Ontario	100.0	28.2	28.2	39	123	17	78	51
Saint-Rémi	Quebec	100.0	8.5	8.5	113	45	135	176	87
Lakeshore	Ontario	96.0	40.2	38.9	151	195	86	96	180
**Repentigny**	**Quebec**	**94.9**	**84.9**	**81.1**	**166**	**162**	**60**	**131**	**168**
(2) ^*^									
Brampton	Ontario	18.5	650.1	148.5	151	210	1	149	127
Vaughan	Ontario	23.3	321.2	102.6	185	217	6	145	179
Cambridge	Ontario	68.3	137.2	96.4	144	137	22	142	131
Pickering	Ontario	87.1	98.6	84.4	182.5	194	11	135	187
**Repentigny**	**Quebec**	**94.9**	**84.9**	**81.1**	**166**	**162**	**60**	**131**	**168**
**(3)**									
Saint-Charles-Borromée	Quebec	4.5	13.7	0.4	1	1	53	2	15
Cowansville	Quebec	16.0	14.3	1.9	2	3	98	1	30
Oakville	Ontario	1.0	212.1	3.5	3.5	9	27	13	217
Mont-Laurier	Quebec	16.0	13.7	1.9	3.5	5	193	16	24
Acton Vale	Quebec	6.7	7.3	0.3	5	7	148	23	20

* CSD appearing among the top 5 in more than one ranking criterion is bolded in the table.

### 3.3. Sensitivity analysis

The spatial distributions of monitor density varied by bandwidth selection methods, as did the definition of our outcome variable in the mixed-effects model (i.e. the bottom quintile). For cross-validated bandwidths, density estimates selected by MSE diminished more rapidly from the monitor sites, leaving large areas with zero density, while likelihood-based density appeared over-smoothed with high quintiles around the most populated cities (figures S11(b) and (c)). Alternatively, increasing the PM_2.5_ gradient percentage cut-off led to converged spatial patterns (figures S11(a) and (d)–(f)), growing proportions of bandwidths reaching the maximum threshold (i.e. outlier mid-point distance between any two monitors) and shrinking number of monitoring gaps identified (table S6). This signalled a greater extent of overlapping monitor spatial coverage.

Compared to Queen contiguity as the spatial weight for the spatial lag term in the mixed-effects model, IDW (within 5 km) and k-NN (*k* = 5) methods had higher AIC (Akaike Information Criterion) values and failed to meet the assumption of spatial independence of residuals from the Moran’s I test (table S7). Estimates and standard errors with k-NN spatial weight were similar to those with Queen contiguity, but using IDW resulted in extremely large intercepts in both socio-economic deprivation and disability models (tables S8 and S9). These might imply local spatial autocorrelations existed at a scale broader than the IDW scheme could effectively capture, which also led to the more negative coefficient of surface PM_2.5_. With k-NN lags, the estimates were relatively stable because most neighbours selected still had shared edges or corners with the focal unit when *k* was small, like below 10.

Despite attaining a lower AIC value when changing the outcome variable to per-unit area monitor density, its p-value in the Moran’s I test was marginally significant (*p* = 0.053) (table S7). Like the IDW spatial lag model, per-unit area monitor density resulted in inflated intercept estimates even when population density was included as a covariate (tables S10–S14), and this spilled over to the large magnitudes of coefficients of surface PM_2.5_ and population density to negate the constant. Since air pollution monitoring is heavily concentrated in areas of pressing need—those with high pollution levels and dense populations—careful examinations are warranted when modelling per-unit area monitor disparity alongside other socio-demographic inequalities. Moreover, associations between CIMD and monitor density became not statistically significant, and the CMA-specific estimates and OR for paediatric physical and psychological disabilities reversed, as compared to the adjusted outcome of per-capita density (table S14).

## 4. Discussion

Our findings highlighted the 5 year PM_2.5_ monitoring gaps and co-occurrence of disparities in PM_2.5_ monitoring and SES deprivation in eastern Canada from 2020 to 2024. Although disability-related disparities were not consistently observed at the regional scale, our province‐ and CMA‐specific estimates revealed localized inequities that would otherwise be obscured in aggregate analyses. Anticipating the update of CAAQS in 2030, we applied locally adaptive KDE with custom bandwidth selection criteria to better represent each monitor’s effective coverage accounting for proximal gradient of surface PM_2.5_, especially for areas close to the exceedance level. This approach allowed us to identify communities with insufficient monitoring capacity and at risk of exceeding air quality standards, where stakeholders can prioritize for expanding PM_2.5_ monitoring, including governmental and community-initiated efforts, to achieve more equitable exposure assessment.

Eastern Canada’s under-monitored communities occurred mainly in suburban areas where a variety of local and regional PM_2.5_ sources emerged. Monitoring gaps in southern Ontario, particularly Essex County, Windsor, and the Niagara municipality, were influenced by both local emissions and long‐range transport from the Ohio River Valley and northern United States (Ontario Ministry of the Environment 2002, Jeong *et al* 2011, Liu and Cui 2014, Sofowote *et al* 2015, Zhang *et al* 2021). Within the Niagara region, the overlap of monitoring gaps and areas with clustered high-emission facilities in Welland and Port Colborne also highlights the inadequacy of current PM_2.5_ monitoring to capture local pollution, posing increased health burdens for the high disability rates and marginalization (figures 1, S5, S7(f), S8(d), S9(d), and S10(d)). Similarly, near Toronto, the largest cluster of under-monitored communities with PM_2.5_ exceeding the 2030 annual CAAQS occurred in northeast Brampton, which is proximal to significant emission sources (airport, highways, warehousing and logistics zones) (figure S7(c)). Southeast Kitchener and Cambridge had under-monitored DAs and CTs near industrial zones and the intersections of Ontario highways 7, 8, and 401 (figures S7(d) and S8(a)). Locations of under-monitored off-island suburbs surrounding Montreal align with the primary highway network, which contributed to traffic-related PM_2.5_ (figure S7(g)). High-quintile areas of satellite-derived surface PM_2.5_ in northwestern Ontario and central-northern Ontario-Quebec boundary were due to the 2021 and 2023 Canadian wildfires (figure S3) (Erni *et al* 2024, Trieu *et al* 2024, Chen *et al* 2025). Increasing wildfire frequency warrants robust PM_2.5_ monitoring to improve emergency preparedness and response throughout wildfire seasons (Wang *et al* 2015). Among under-monitored DAs that did not exceed the 2030 CAAQS but did exceed the 2021 WHO guideline in Quebec (figures S7(h) and S7(i)), Thetford Mines has historically experienced air pollution due to mining activities (Belleville 2011), while municipalities including Sainte-Marie and Saint-Georges have sustained growth in manufacturing activities, giving rise to environmental pollution (Guillaume and Doloreux 2011).

Marginalized communities prone to exceeding the new air quality standard should be prioritized in future PM_2.5_ monitoring. A significant number of suburbs near Toronto and Montreal were found to exceed the 2030 annual CAAQS (figures S7(c) and (g)). Previous PM_2.5_ monitor deployments in Toronto and Montreal were concentrated in downtown areas. A relocation of PM_2.5_ monitors, particularly low-cost sensors, in these two largest metropolises may help to reduce the imbalanced ratio between central-urban and suburban monitoring capacity. Furthermore, prior research using CIMD showed that areas with higher marginalization scores experienced poorer health outcomes (Carino *et al* 2021, Goobie *et al* 2022, Antonova *et al* 2023) and disparities in healthcare access (Wenghofer and Ransom 2023). Siting of monitoring expansion should also include communities with combined disparities in SES, population health vulnerability, and PM_2.5_ exposure. We identified this type of communities near Kitchener, Cambridge, Niagara, and Oshawa (figures S7(c), S7(d), S7(f), S8(a), S8(b) and S8(d)). In Canada, mental health-related disabilities are more common among children while physical limitations are more prevalent in the elderly (table S15) (Statistics Canada 2023c). Exposure and monitoring assessments are recommended to target these two groups, particularly in Toronto, Montreal, Niagara, and Oshawa, where monitoring disparities were more pronounced (tables S3 and S4). Under-monitoring of PM_2.5_ can increase burdens of local healthcare system due to the elevated risk of emergency department visits for paediatric mental disorders (Szyszkowicz *et al* 2020) and higher hospitalization rate among people with disabilities or pre-existing health conditions (Park *et al* 2024). The two U.S.-Canada cross-border regions of concern, Niagara and Windsor, have larger groups of lower-income, disabled populations and retirement communities that would be disproportionately affected by air pollution health effects and are less likely to be able to relocate during severe-pollution events. Specific to Niagara, geographic distance can be a barrier for access to primary healthcare (Lum *et al* 2016), which would exacerbate disadvantages of people with disabilities in smaller towns. Experience from U.S.-Mexico border health studies suggested that community-led cross-border collaboration and community-rooted intervention measures, such as rapid emergency aid and workforce coordination, can empower these communities to build stronger resilience to environmental health susceptibility (Galdámez *et al* 2025).

Observed PM_2.5_ monitoring disparities among SES-deprived populations might be associated with the socio-spatial geography in densely populated areas. The observed lower availability of PM_2.5_ monitors among marginalized groups in residential instability and situational vulnerability (table 1) might be linked to demographic shifts driven by recent Canadian migration and immigration patterns characterized by increasing movements from urban centres to peripheral and rural communities—where PM_2.5_ monitoring is sparse—for affordable housing (Vézina and Houle 2017, McQuillan 2024). Suburban and remote communities may also have limited funding to utilize low-cost sensors and were of a lower priority in past regulatory monitoring site selection. With densification of housing supply and expansion of urban sprawl neighbourhoods (Pârvulescu *et al* 2024), there is a need to augment the PM_2.5_ monitoring capacity in these fast-growing areas to address changes in population health geography and ensure effectiveness of future air pollution management measures. Moreover, the significance of economic dependency and ethno-cultural composition for Ontario and NB, respectively, implies the importance of tailored approaches within provinces, and even at intra-municipality scales. Economic dependency varies according to proportions of employed individuals against youth and senior dependents. Ontario has a workforce concentrated in the major economic hubs of Toronto and Ottawa, indicating a high inequality marked by the highest Gini coefficient among eastern provinces in 2021 (Statistics Canada 2023e, 2023f). Neighbourhoods with lower employment accessibility, such as Niagara, Windsor, and London, often have older populations (Statistics Canada 2022b), which coincide with the monitoring gaps we found in Ontario. Older adults also tend to live in rural areas or isolated clusters in Toronto (Channer *et al* 2020), hence monitoring strategies must be adaptable and community-oriented. Our analysis also hints at inadequate PM_2.5_ monitoring in communities with higher proportions of physically-disabled children within each province and CMA, despite being obscured in the fixed effect estimate. Recent evidence established a positive association between air pollution exposure and both a progression toward more severe disability states and a reduced likelihood of recovery among ageing cohorts (Weuve *et al* 2016, Gao *et al* 2026). Similar patterns may also apply to paediatric populations, but less research has been conducted on the progression of physical function limitations among children living with disabilities, compared to that on the incidence of adverse health outcomes, such as asthma and depression, associated with exposure in prenatal or early-life phases (Roberts *et al* 2019, Aithal *et al* 2023). Despite high uncertainty in random intercepts, results from unadjusted absolute monitor density might suggest monitoring inequalities for children with psychological disabilities in several mid-sized cities like Fredericton, NB; Quebec City and Trois-Rivières in Quebec; London, Sudbury, and Sault Ste. Marie in Ontario (table S14). Overall, our results point to air pollution monitoring gaps that could be leading to exposure data constraints when researching the health effects of PM_2.5_ exposure among marginalized and vulnerable population groups.

There are limitations in this study. First, there is no absolute criterion for local bandwidth selection. Generally, we wanted to avoid over-smoothing and underestimation in coverage. Although cross-validation subject to an error function of choice could empirically optimize to the shape of distributions, it might not be practically applicable for our analysis. MSE cross-validated estimates rendered large remote areas as monitoring gaps, and this could not distinguish low-capacity areas that had pollution levels representable by proximal monitors (figure S11(b)). Meanwhile, likelihood cross-validated estimates produced high urban-rural contrasts, which would easily hide monitoring gaps near densely populated areas and neglect remote neighbourhoods with monitoring efforts (figure S11(c)). In this case, if we used the bottom quintile as the outcome variable, it might exaggerate disparities with ethno-cultural minorities and situationally vulnerable groups, who are more populated outside of urban centres (figure S6). In the context of environmental resource management, we usually consider thresholds to inform decision-making. We based our selection on the new CAAQS to identify priority areas for monitoring actions. The buffer percentage differences in PM_2.5_ levels as bandwidth cut-offs were positively linked to urban-rural monitor density variations (figures S11(a) and (d)–(f)). Thus, higher cut-offs would have similar drawbacks as the likelihood cross-validated estimates. Still, this choice may vary with different areas of interest. Second, comparison using census units could encounter the modifiable unit area problem (Buzzelli 2020), which means the conclusion of disparities might be sensitive to the boundary shapes and sizes. Within a unit, locations with zero monitor density might be omitted from unit-wide average. For intra-urban or intra-provincial comparisons, regional CIMD indicators might be more suitable (Statistics Canada 2019). However, the included census variables and their principal component loadings in the four dimensions can differ from the national CIMD, thus yielding different conclusions. Third, the ACAG’s model was trained on available ground-based observations, which could lead to higher uncertainties for surface PM_2.5_ estimates in sparsely monitored areas. Still, the model demonstrated robustness in performance when up to 80% of sites were withheld for testing (Shen *et al* 2024). Lastly, our study period covered all deployments during 2020–2024, and did not investigate annual variations. Effective policy planning should also consider population dynamics, industrial development, and climate change that can alter air pollution and socio-demographic marginalization distributions. Finance and public acceptance are other constraints for actionable framework. Moreover, unlike common accessibility measures on healthcare, transport, employment, etc., resource capacity for air pollution surveillance does not have a widely established baseline for pinpointing policy interventions. Research is needed to understand what levels of monitoring capacity, in counts, density, or other analytical indicators, would best guide the strategies to reduce biases in population exposure estimates, especially when low-dose exposure contributes to elevated mortality risk (Weichenthal *et al* 2022). While we found the distance-based approach ineffective for modelling spatial dependence in our study, another study based in Rio de Janeiro, Brazil compared neighbourhood pollution and demographic trends by categorizing distances from monitors and found social deprivation, paediatric populations, and ozone concentration were increasing with distance within the first 5 km from monitors (Kim *et al* 2024). This reiterated that there is no one-size-fits-all evaluation strategy. Therefore, future efforts should involve systematically reviewing data gaps, tracking changes in community needs, and employing mixed research design like participatory research to bridge the gaps between resource management and community health.

Our study showcases how open science data can be harmonized to address an environmental health research gap and a practical challenge for community-engaged PM_2.5_ monitoring. As utilization of low-cost sensors potentiates high-resolution surface exposure assessment, their deployments require an efficient, affordable, and easy-to-implement framework to optimize the representativeness to promote equity in social and health benefits. The ACAG’s PM_2.5_ model (SatPM_2.5_) data and AQMap archive of PurpleAir measurements are publicly accessible. Specifically, SatPM_2.5_ incorporated different factors that the government considered for regulatory monitor siting, such as meteorology, background emissions, and elevation, into model training (Shen *et al* 2024). This provides a reference for initial siting decision of low-cost sensor deployments and reduces the time and costs for field sample collection and analysis. The locally adaptive KDE built on this satellite-derived data is also a flexible and assumption-free statistical approach to calibrate the monitor’s representativeness in neighbourhood accounting for local PM_2.5_ gradient. Decision-makers can manipulate individual bandwidths to fit their own monitoring objectives. In practical applications, we can simulate hypothetical addition of monitors in prioritized areas and assess the changes in monitoring density under different scenarios. Since PurpleAir has moved to a paid service for data access, sustained collaborative efforts and support are critical for facilitating monitoring information sharing, particularly for informing public health risk. For future analysis, a thorough evaluation of PM_2.5_ monitoring network can integrate multiple metrics (e.g. monitor density, monitor operating consistency, non-attainment rate in capturing PM_2.5_ exceedances, co-location and calibration performance of low-cost sensors).

## 5. Conclusion

In summary, this study offers insights into the PM_2.5_ monitoring gaps and disparities in eastern Canada. Using neighbourhood variations in satellite-derived surface PM_2.5_ concentrations at a high spatial resolution, we adopted locally adaptive KDE to estimate the monitor density incorporating FEM stations and PurpleAir sensors. We highlighted under-monitored areas characterized by having PM_2.5_ exceedances under the 2030 CAAQS or current WHO guideline, and facing social disadvantages or health vulnerability. Future monitoring and exposure assessments should focus on suburban communities of Montreal, Toronto, near-border regions of Windsor and Niagara, industrial-adjacent communities in southeast Kitchener, and remote communities in northwest Ontario. In general, residentially insecure and situationally vulnerable populations were underserved in PM_2.5_ monitoring. Paediatric populations with disabilities had lower share of monitoring resources within local scales (provinces or municipalities). Future monitoring policy and community-driven programmes need to account for identified inequalities to improve estimations of health risks and surveillance of PM_2.5_ exceedances.

## Data Availability

The data that support the findings of this study are openly available at the following URL/DOI: https://www.arcgis.com/home/item.html?id=a3f8cfe467ef4c5687d94757bae6909c for the mapping results on ArcGIS Online (Siu 2026b); and https://github.com/tksiu/pm25_monitor_disparities for the codes available on GitHub that were used to implement the analysis (Siu 2026a). Supplementary Information available at: https://doi.org/10.1088/2752-5309/ae87c0/data1.
